# Perceptions of Chinese women with a history of gestational diabetes regarding health behaviors and related factors: a directed qualitative content analysis

**DOI:** 10.1186/s12889-024-18731-3

**Published:** 2024-05-06

**Authors:** Xiaoxia Ma, Yun Yang, Shuhua Qian, Yan Ding, Qiping Lin, Na Wang

**Affiliations:** 1 Nursing Department, Obstetrics and Gynaecology Hospital of Fudan University, No. 128 Shenyang Road, Shanghai, 200090 China; 2 School of Nursing, Fudan University, Shanghai, China

**Keywords:** Diabetes, gestational, Type 2 diabetes mellitus, Health behavior, Qualitative research, Theory of planned behavior

## Abstract

**Background:**

Gestational diabetes mellitus (GDM) is one of the most common metabolic disorders during pregnancy and is associated with adverse outcomes in both mothers and their children. After delivery, women who experience GDM are also at higher risk of both subsequent GDM and type 2 diabetes mellitus (T2DM) than those who do not. Therefore, healthcare providers and public health practitioners need to develop targeted and effective interventions for GDM. In this study, we aimed to explore the perceptions regarding health behaviors and related factors during the inter-pregnancy period among Chinese women with a history of GDM through the lens of the theory of planned behavior (TPB).

**Methods:**

Between December 2021 and September 2022, 16 pregnant Chinese women with a history of GDM were purposively recruited from a tertiary maternity hospital in Shanghai for face-to-face semi-structured interviews. They were asked questions regarding their health behaviors and related factors. The transcribed data were analyzed using a directed qualitative content analysis method based on the theory of TPB.

**Results:**

The health-related behaviors of the women varied substantially. We identified five domains that influenced women’s behaviors according to TPB constructs and based on the data collected: behavioral attitude (perceived benefits of healthy behaviors and the relationship between experience and attitude towards the oral glucose tolerance testing); subjective norms (influences of significant others and traditional cultural beliefs); perceived behavior control (knowledge of the disease, multiple-role conflict, the impact of COVID-19, an unfriendly external environment and difficulty adhering to healthy diets), incentive mechanisms (self-reward and external incentives); preferences of professional and institutional support (making full use of social media platform and providing continuous health management).

**Conclusions:**

The health-related behaviors of women with a history of GDM were found to be affected by multiple factors. Healthcare professionals are recommended to provide women with sufficient information regarding the disease and to take advantage of the power of the family and other social support networks to improve women’s subjective norms and to promote the adoption of a healthy lifestyle.

## Background

Gestational diabetes mellitus (GDM) refers to impaired glucose tolerance that is first identified during pregnancy [1], and is increasingly prevalent worldwide. It is estimated that 21.1 million (16.7%) of live births to women in 2021 were associated with some form of hyperglycemia during pregnancy [2]. Of these, 80.3% cases were the result of GDM [2], which is associated with adverse outcomes in both perinatal women and the fetus, including preeclampsia, emergency caesarean section, macrosomia, and premature birth [3]. After delivery, mothers who experience GDM are at a tenfold higher risk of type 2 diabetes mellitus (T2DM) and the recurrence of GDM in a subsequent pregnancy than those who do not [4, 5]. Besides, the exposure of high glucose environment within the uterus leads to increased risk of long-term metabolic diseases such as obesity, diabetes and cardiovascular disease in offspring [6, 7].

Postpartum health behaviors, and especially diet and physical activity levels, are strongly associated with the risk of diabetes. The Da Qing Impaired Glucose Tolerance and Diabetes Study [8], which was performed in China, showed that diet and/or exercise interventions led to significant reductions in the incidence of diabetes over the following 6-year period in women with impaired glucose tolerance. A similar study performed in the United States, the National Diabetes Prevention Program [9], which had the goals of a ≥ 7% weight loss and 150 min of physical activity per week, reduced the incidence of diabetes in patients at high risk by 58%. According to the statement position of the American Heart Association [10] and the American Diabetes Association [11] on postpartum and interpregnancy management of GDM, women should undergo oral glucose tolerance testing (OGTT) 4–12 weeks postpartum, weight loss and exercise for diabetes prevention, etc. However, following childbirth, most women do not make the appropriate lifestyle adjustments or undergo OGTT as recommended. Studies performed in China and other countries have shown that attendance at a clinic for glucose screening is undesirable in women who have recently experienced GDM [12–14]. In fact, only 58% of women were found to take up the offer of glucose screening during the first year following delivery and the percentage attending declined substantially in the second and third years [15]. Furthermore, women who experience GDM tend to return to their unhealthy lifestyle, have irregular meal patterns, exercise at lower intensity, and gain more body weight than women who remain normoglycemic during pregnancy [16, 17].

In real-world settings, changes in health behavior are difficult to maintain. Studies have suggested that women who have previously experienced GDM face multiple barriers to the maintenance of healthy behaviors following delivery, including a lack of knowledge regarding diabetes risk, insufficient social support, negative emotions, and prioritization of the needs of their family [18–20]. Therefore, effective interventions that take into account these barriers and involve tailored solutions should be developed. In China, the government has gradually relaxed fertility restrictions from the one-child policy to universal two- and three-child policies. With these changes in family planning policy, an increasing number of women have become pregnant at a more advanced age, along with a high body mass index and a history of GDM, which have led to increases in the incidences of pregnancy complications and comorbidities [21, 22]. However, to date, evidence regarding the prevention of recurrence of GDM through comprehensive lifestyle interventions is scarce. And few studies have applied implementation science and behavioral change theories to develop and implement interventions that enable sustained behavioral changes in the target population; for example, women with a history of GDM in China, which has unique culture and healthcare systems.

The aim of the present study was to explore the perception regarding health behaviors and related factors during the inter-pregnancy period among Chinese women with a history of GDM through the lens of the theory of planned behavior (TPB). TPB suggests that behavioral intention determines individual behavior, which in turn is influenced by behavioral attitudes (the positive or negative evaluation of a particular behavior), subjective norms (the influence of important others or groups on individual decisions) and perceptual behavior control (perception of the ease or difficulty of a particular behavior) [23]. It has been well developed and proven to have strong explanatory power and predictive ability for health behaviors, widely used in areas such as physical activity and diet behaviors [24, 25].

## Methods

### Design and setting

We performed a qualitative study that used in-depth interviews to explore the perceptions and influences on inter-pregnancy health-related behaviors, of women who had experienced GDM and who attended a tertiary maternity hospital in Shanghai, China, between December 2021 and September 2022. Shanghai is the largest city in China and the study hospital is a university-affiliated hospital at which there were ~ 12,000 births annually over the preceding 5 years.

### Participants

Pregnant women with a history of GDM were selected at the hospital to obtain a wide range of maternal ages and numbers of gestational weeks. The participants were recruited according to the following eligibility criteria: a diagnosis of GDM during the previous pregnancy, based on the International Association of Diabetes and Pregnancy Study Groups criteria published in 2010 [26]; age ≥ 18 years, and fluency in Mandarin Chinese. Women with mental or cognitive impairment were excluded from the study. Briefly, women who attended their first routine antenatal care session at the hospital were approached by a trained nurse, who considered their eligibility, and they were then referred to two interviewers (Na Wang and Xiaoxia Ma).

Table 1 shows the sociodemographic characteristics of the participants. In total, 16 pregnant women, ranging in age from 30 to 43 years, who had a history of GDM during their previous pregnancies, were interviewed between December 2021 and September 2022. The gestational intervals of the participants ranged from 2 to 8 years. Half of them had been administered insulin during their previous pregnancies. After giving birth on the previous occasion, only three of the participants (P7, P12 and P16) had undergone OGTT 4–12 weeks postpartum, as recommend by the guidelines, two had been identified as being hyperglycemic when they visited a hospital for another reason 1–2 years postpartum (P5 was diagnosed with diabetes and P14 was diagnosed with insulin resistance), and five participants had their fasting blood glucose or glycosylated hemoglobin levels measured when they attended the routine physical examinations organized by their units, 1 year postpartum, and the remaining six women did not have blood glucose concentrations measured during the inter-pregnancy period at all. Most of the participants (87.5%, 14/16) had experienced recurrence of GDM during their present pregnancy.

**Table 1:** Sociodemographic characteristics of the participants

No	Age (years)	Education background	Family history of T2DM	Treatments during previous pregnancy	Postpartum lifestyle interventions	Postpartum OGTT	Gestational intervals (months)	Recurrence of GDM in the present pregnancy	Gestational weeks during the interview (wks)
P1	31	College	Yes	A + B + C	A	No	24	Yes	12
P2	34	≥ Postgraduate	Yes	A + B + C	A	No	25	Yes	13
P3	36	College	No	A	A + B	No	62	Untested	10
P4	34	College	No	A + B	—	No	78	Yes	17
P5	43	College	No	A	—	Yes	69	Yes^a^	39
P6	32	≥ Postgraduate	No	A + B	—	No	53	Yes	34
P7	30	College	No	A + B	B	Yes	46	No	39
P8	35	College	Yes	A	—	No	36	Yes	35
P9	32	College	Yes	A + B + C	A + B	No	65	Yes	10
P10	35	College	Yes	A + B + C	B	No	36	Yes	14
P11	34	College	Yes	A + C	B	No	36	Yes	39
P12	30	College	Yes	A + B + C	B	Yes	48	Yes	40
P13	35	College	No	A + B	A + B	No	72	Yes	40
P14	33	College	No	A + B	B	Yes	30	Yes	10
P15	33	≤ Senior high school	No	A + B + C	A + B	No	66	Yes	39
P16	35	College	Yes	A + B	—	Yes	96	Yes	38

A. Dietary controlled, B. Exercise regularly, C. Insulin therapy

^a^P5 had pre-existing T2DM in her present pregnancy rather than a de novo diagnosis of GDM

The study was approved by the hospital Ethics committee (approval no. 202123), and all the participants provided their written informed consent to be interviewed. The sample size was selected based on the principle of saturation of qualitative research data, and 16 women were finally interviewed.

### Data collection

On the basis of the purpose of the present study and a literature review of related studies, semi-structured interview guidelines were developed and used to conduct in-depth interviews. The interview consisted of a standardized set of open-ended questions that focused on the health-related behaviors of women who had previously experienced GDM, which were followed by further probing questions. Two women participated in individual pilot interviews to test the efficacy of the interview procedure before the official interviews. The final list of questions was as follows:

1. What do you know about this disease?
2. What do you think about the impact of GDM on your health in the future?
3. How does it affect your daily life? What changes have you made to your daily life after being diagnosed with GDM?
4. How about your lifestyle after giving birth? Did someone give you some advice postpartum regarding lifestyle issues, such as diet and exercise, or blood glucose screening, relating to GDM?
5. What are the challenges you face or the facilitators of the maintenance of a healthy lifestyle following your last birth or during the inter-pregnancy period?
6. What support, especially from health professionals, do you expect after giving birth? At what time and in what form?

Before starting the interview, the participants were asked to complete a short questionnaire regarding their age, educational background, family history of diabetes, treatments during previous pregnancy, postpartum lifestyle interventions, attendance for postpartum glucose screening, gestational intervals, a diagnosis of GDM during the present pregnancy, and their number of gestational weeks at the time of the interview. After building a rapport, we started the interview with the broad question “What was your experience of GDM in your last pregnancy”? When describing their experiences, participates often talked about the advice given by the medical staff upon diagnosis with GDM along with changes in their lifestyles. Followed by questions further involved their understanding regarding the disease and its impact on their postpartum lifestyles. If they have continued a healthy lifestyle after giving birth, e.g., healthy diet, regular exercise, etc., a follow-up question regarding promoting factors was asked; if not, we further investigated what the barriers were and what support they needed. The interviews were conducted in Mandarin Chinese and field notes regarding the participants’ expressions, emotions, and behaviors during the interview were also taken to facilitate subsequent data analysis.

The interviews were conducted by two well-trained interviewers and audio-recorded. They lasted between 25 and 60 min. We recruited a final sample size of 16 women for data saturation, which means no further information relevant to the research was introduced. Each participant was offered a 20-yuan gift as compensation for their time and cooperation.

### Data analysis

Audio recordings were transcribed verbatim, word-by-word, within 24 h, and imported into the qualitative analysis software NVivo 12.0. Adhering to the suggestions provided by Satu Elo [27], the data analysis process consists of the following steps: (1) selecting the units of analysis and being immersed in the data to gain a holistic understanding of women’ descriptions; (2) developing a categorization matrix deductively derived from TPB (behavioral attitudes, subjective norms, and perceptual behavior control) [23]; (3) coding the data according the pre-determined categories; (4) besides, through the inductive coding, the new categories that did not fit within the TPB domains emerged. The combination of inductive and deductive approaches used for the data analysis facilitates more synergistic findings to be made regarding the influences on and determinants of women’s behaviors [28]. The data analysis was conducted concurrently with the data collection, to determine whether new codes and categories emerged during the interviews. To ensure the rigor of the study, two researchers independently reviewed and analyzed the transcripts and field notes. Any discrepancies in the data analysis between the two researchers were resolved through discussion.

## Results

**Fig. 1:**
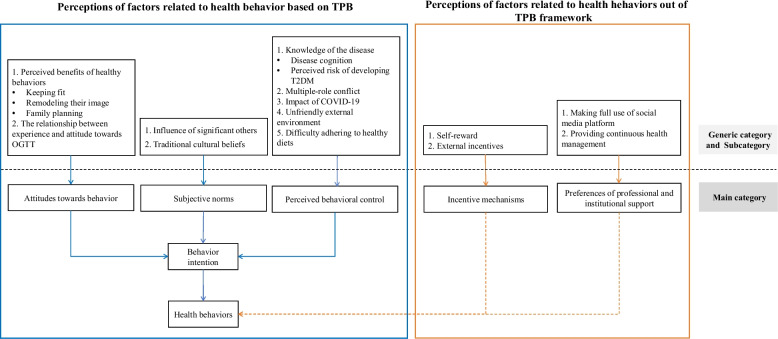
Perceptions of Chinese women with a history of gestational diabetes regarding health behaviors. The orange dotted lines indicate factors related to health behaviors that were beyond the theoretical domain of TPB in this study, which have not been rigorously validated in other populations

The health-related behaviors of the women varied substantially. After careful review of the text, through deductive approach, three categories of related factors were identified that fit the structures of TPB. Additionally, two other categories that did not fit TPB frame (incentive mechanisms and preferences derived from professional and institutional support) were generated using the inductive approach. As presented in Fig. 1.

### Attitudes towards health-related behavior

#### Perceived benefits of healthy behaviors

##### Keeping fit

Owing to their irregular eating habits and physical inactivity, 3 women (18.75%) developed cardiovascular diseases following their previous birth, which made them realize that they had to take responsibility for their own health by adopting a healthy lifestyle.

*P7: “Six months after the birth, I went for a routine physical examination. The doctor said that I had gained too much weight, almost 25 pounds. Then I was diagnosed with…uh…fat infiltration into my liver, which made me pay more attention to my diet and exercise… I did yoga for about 20 min each evening and I lost a lot of weight.”*

*P13: “There were a lot of problems at work… and I had to take care of the child after work. It was very stressful (sigh). So, I chose to do yoga, during which I can be alone and relax my mind.”*

##### Remodeling their image

To get slim is a powerful impetus for some women to follow a healthy diet and be physically active.

*P10: “In order to lose weight and look beautiful, I insisted on taking a brisk walk for an hour every day, unless it was rainy, and I also went swimming once a week at that time. Besides, I subconsciously balanced my diet, by controlling my sugar intake, eating more vegetables, and reducing the amount of high starchy food I consumed… I lost 20 pounds in 5 months! (loudly).”*

*P11: “I will return to work after my maternity leave and I must prioritize my body image… which is very important.”*

##### Family planning

Women who plan to have a second child were more likely to have a positive attitude toward glycemic control and to adopt the recommendations regarding the maintenance of a healthy lifestyles before conception.

*P13: “After giving birth to my first baby, I planned to have a second one. I thought it was necessary to control my staple food intake and reduce my sugar intake. Therefore, coarse grains, taro, and sweet potatoes were my first choices for every meal.”*

In contrast, some participants said that the second pregnancy was accidental, and therefore they did not make any preparations during the early stages, which might have prevented them from maintaining a healthier lifestyle.

*P6: “To be honest, I did not plan to have another baby and I haven’t adjusted my lifestyle significantly during the past three or four years… The recurrence (of GDM) was… not surprising.”*

#### The relationship between experience and attitude towards OGTT

The previous experience of the unpleasant taste of the glucose syrup and the repeated venipuncture during OGTT appeared to affect women’s attitude to undergo this procedure again following delivery.

*P2: “At that time, he (the doctor) reminded me to do OGTT after delivery, but I remembered that the process was painful. I put it off as long as I could and in the end I did not do the test.”*

*P5: “The sugary water (consumed during OGTT) was too sweet to drink, so I didn’t want to drink it again.”*

### Subjective norms

#### Influence of significant others

The “significant others” in the lives of the participants were principally family members, colleagues, and health professionals. The influence of their family was identified as a key determinant in the establishment of a healthy lifestyle by women with a history of GDM.

*P14: “My husband sets a great example for me because he is a fitness fanatic, which also influences me. In addition, my parents attach great importance to health, and they urge me to make changes every day, such as ‘Why don’t you go running? It’s time to exercise’ (laugh).”*

*P2: “My husband is very lazy; he always lies on the couch playing with his mobile phone… I must ask someone else if I want to play badminton, which makes me lack motivation to exercise… Uh…in fact, I don’t like exercise myself. None of us like exercise.”*

In addition to this, the attitudes and behavior of the women’s friends and colleagues also had an influence on their behavior.

*P12: “Experiencing the physical examination organized by the unit in recent years, all of my colleagues feel that one’s health is very important… We have been playing badminton together for the last 2 or 3 years to strengthen our bodies.”*

Notably, the health professionals at the maternity hospital were the most authoritative and important influences on the women with GDM regarding the risks of T2DM following GDM and the importance of blood glucose screening and maintaining a healthy lifestyle postpartum. These were in the form of warnings and urging.

*P7: “When I was discharged from the hospital, the doctor told me that if I didn’t control my blood sugar after birth, I might develop permanent diabetes. I am worried about it, so I continued to eat a balanced diet after birth. And he also advised me that it was better to test my blood sugar by… drinking sugary water (OGTT) or… something I don’t remember any more, to prevent diabetes. It (diabetes) was likely to continue and affect my later life.”*

Despite these responses, most of the women interviewed in the present study reported that no-one had reminded them about maintaining a healthy lifestyle or undergoing blood glucose screening following birth. They had poor awareness of the long-term implications of GDM.

*P2: “The doctor didn’t say anything about a relapse of the disease or the probability of diabetes in the future… I was never told before (by the doctor)”.*

#### Traditional cultural beliefs

The observance of the traditional cultural “confinement” custom limits women’s dietary choices and activities during the first month following delivery, and these limitations can continue well into their lactation period.

*P10: “When I breastfed my baby after childbirth, my mother-in-law made me drink all kinds of soup every day, such as fish soup, chicken soup and so on, in order to produce more milk; and I was not allowed to go out in the period after giving birth. All I could do every day was to eat, sleep, feed, and eat again.”*

*P11: “I put on a lot of weight after I gave birth. I breastfed my baby, and the internet and my family and friends said that it is best not to do exercise during the period of breastfeeding… To be honest, I do not understand why, but I started to exercise and lose weight after weaning.”*

### Perceived behavioral control

#### Knowledge of the disease

##### Disease cognition

In the interviews, most women regarded GDM as a temporary abnormality in glucose status. They had poor awareness of the long-term implications of the disease, and rarely realized the importance of either a healthy lifestyle or blood glucose screening following delivery. Knowledge gaps translated into a lower likelihood for them to establish a healthy lifestyle.

*P5: “After birth, I quickly resumed my old lifestyle habits, especially with respect to eating fried foods, which I like very much (laugh). This was because I thought that this disease only happened during pregnancy, uh… and my baby was born very healthy; he was not macrosomic. It (GDM) had no effects on him. The doctor just wanted to scare me.”*

*P14: “Anyway, after giving birth, I didn’t care about it (blood sugar) anymore and did no exercise either… However, about 1 year ago, I felt a little dizzy. Then I was diagnosed with high blood pressure and high blood sugar… It is very strange: no one in our family has diabetes… Is it because I am fat? Now, I am really worried about myself and my baby (sobbing).”*

##### Perceived risk of developing T2DM

Women were more willing to change their sub-optimal lifestyle if they recognized that GDM might increase their risk of metabolic disease, and especially if they were concerned about developing T2DM in the future. 5 women (31.25%) remained a healthy lifestyle for this reason in the postpartum period. This was more evident among women with a family history of T2DM.

*P15: “After giving birth, I attached great importance to my lifestyle. You know, I didn’t even dare to eat porridge anymore- I was really strict- because my doctor told me that I was susceptible to T2DM after this (GDM). I must take responsibility for my own health.”*

*P2: “My grandmother used to have severe diabetes and injected insulin… Unfortunately, she was involved in a car accident a few years ago and her injury did not heal easily… Then she died after that… My uncle also had diabetes, and he experienced retinal bleeding because of diabetes. It scared me! I was worried about whether I would get it in the future.”*

#### Multiple-role conflict

The social roles of women can be categorized as worker, spouse, parent, and daughter. Women need to take care of their newborns and adapt to their new roles as mothers during the early postpartum period. In China, the mothers-in-law of women are the most likely people to help with the care of the children, and sometimes they live with their daughters-in-law. These multiple roles make it difficult for mothers to focus on themselves.

*P7: “Uh… It was totally different having and not having a child. Previously, I might have gone to play ball or do some other sports after finishing work, and it didn’t matter if I went home early or late. Now, the first thing I do after work is rush home to look after my baby (laugh). It is completely different!”*

*P11: “At that time, I was living with my parents-in-law. Uh… There were a lot of conflicts between us, which meant that I was always in a bad mood. I thought it was postpartum depression. And I had to take care of my child and deal with an intense job at the same time… It was too hard (sigh).”*

#### Impact of COVID-19

During the COVID-19 pandemic, Shanghai underwent a lockdown for approximately 2 months, which limited people’s outdoor activities and their attendance at gyms, as well as the variety of food available.

*P3: “I used to go to the gym three times a week. However, owing to the COVID-19 epidemic, I could not go out to exercise and I gained more than ten pounds at that time. It shocked me!”*

*P5: “We were locked down at home because of the COVID-19 pandemic. We couldn’t buy whole grains, or various vegetables or fruits from the market, and had to accept a limited range of food.”*

#### Unfriendly external environment

Despite growing concerns regarding obesity and diabetes during recent years, provision of healthy meals in public places is still limited, which makes it more difficult for such women to pursue a healthier lifestyle.

*P1: “I think the outside environment is not friendly to diabetic people. When eating out, it’s difficult for me to get specific meals.”*

*P6: “It’s very difficult to bring food every day when I go to work, so I eat in the cafeteria. And in that situation, my sugar, oil, and staple food intake can’t be strictly controlled.”*

#### Difficulty adhering to healthy diets

Owing to the relative lack of flavor of “healthy food”, some women find it difficult to observe a healthy diet.

*P4: “After giving birth, I didn’t deliberately maintain the eating habits recommended by the doctors during pregnancy, especially with respect to coarse grains: I couldn’t stand the taste of them. Really, they are terrible; don’t you think so?”*

*P12: “Compared to low-sugar diets, foods such as bread, cakes, and glutinous rice products are more tempting to me. They are my favorites (laugh).”*

### Incentive mechanisms

#### Self-reward

Obtaining a reward is considered to be an important contributor to compliance with an exercise regimen.

*P10: “For me, I need to give myself a little reward to encourage me to keep moving. For example, if I swam for 1 h today, I would reward myself with a cup of low-sugar milky tea (laugh). In this way, I can keep doing exercise more easily.”*

#### External incentives

Some platforms use incentive mechanisms to motivate their customers.

*P14: “Do you know the Keep, the sports software? It can track and record the duration of exercise and display the number of calories I have burned. If you keep moving, you get some cute medals. On that platform, some people upload their fitness dynamics, which is very motivating for me… their figures are so charming!”*

### Preferences of professional and institutional support

#### Making full use of social media platform

During the interviews, some women expressed a need for education by qualified health professionals over the internet, involving videos that improve their knowledge of diabetes following delivery and encourage them to undergo blood glucose screening.

*P13: “I think there will be some promotional videos, which will tell me what I need to pay attention to when I go home. This method is more vivid and impressive.”*

*P2: “For example, if I haven’t visited for OGTT at about 42 days postpartum, I hope that my doctor will remind me and send messages about the risks of missing screening via telephone or WeChat.*

#### Providing continuous health management

Regarding the timing of the support provided by healthcare workers following delivery, most women expected to obtain guidance prior to their discharge from hospital. In addition, during the routine examination of women 42 days following delivery, health professionals can provide education and guidance according to the results of OGTT, and continue to provide support through an online platform.

## Discussion

In the present study, we interviewed 16 Chinese women with a history of GDM to explore their health behaviors and related factors through the lens of TPB theory, during the inter-pregnancy period. We found that their behaviors varied substantially, and most were not optimal. Further exploration of the determinants of their behaviors revealed that in addition to some personal characteristics, a lack of awareness of the risks of diabetes, multiple-role conflicts, insufficient social support, and traditional cultural beliefs were repeatedly mentioned as factors that had negative impacts on the pursuit of a healthy lifestyle. In addition, some of the barriers were perceived to be beyond their control. In contrast, women who adhered to a healthy lifestyle believed that the incentive mechanisms and sufficient social support had positive impacts on their performance.

With the economic development and lifestyle changes occurring around the world, the prevalence of obesity is gradually increasing, and women of child-bearing age are no exception to this [29]. Obesity is associated with a range of health problems, including diabetes, hypertension, hyperlipidemia, coronary heart disease, and other chronic diseases, and weight loss can significantly reduce the risk of obesity-related diseases [30]. In the present study, women who developed conditions associated with obesity during the inter-pregnancy period were more willing to maintain good physical condition through lifestyle improvements, such as increases in physical exertion to achieve weight loss and a reduction in the consumption of foods that are rich in fat and sugar. In addition, the desire to improve body image is an important intrinsic motivation that increases the likelihood of women adopting a healthy diet and higher physical activity levels.

Of note, we found that women who were willing to prepare for their subsequent pregnancies were more likely to adopt a healthy lifestyle. However, a previous study showed that the unintended pregnancy rates, both in China and worldwide, remain high [31]. In the present study, only two of the women had a reproduction plan. A retrospective cohort study conducted in the United States showed that people who planned their pregnancies were less likely to smoke before becoming pregnant, were more likely to regularly take multivitamins, and were more likely to have undergone health checks during the preceding year [32]. In contrast, women with an unplanned pregnancy had higher body mass indexes and were less likely to take a folic acid supplement [33]. According to the guide “Optimizing Postnatal Care”, patients and their obstetricians/gynecologists or other obstetric care providers should discuss the woman’s reproductive life plans, beginning during prenatal care, and including a discussion regarding future pregnancies and their timing [34]. Therefore, medical staff in maternity hospitals may need to highlight the need for postpartum contraception and discuss future maternity plans with women who experience GDM and their partners, and encourage them to adopt healthy behaviors.

Furthermore, OGTT is time-consuming, involves the ingestion of unpalatable glucose syrup, and requires multiple blood samples to be collected. Therefore, this can be an unpleasant experience for women [35], and they may be reluctant to undergo this process again following delivery. As healthcare professionals, we should raise women’s awareness of the importance of postpartum OGTT and increase the rate of screening through telephone, emails and other internet platforms [36, 37].

Advice from health professionals is often thought to be the most important means of improving women’s knowledge regarding diabetes and the benefits of maintaining a healthy lifestyle postpartum. However, previous studies have shown that the information women receive regarding postpartum glucose management is too limited. A study conducted in Singapore showed that although women typically receive advice regarding healthy lifestyles from medical staff, this largely focuses on the prevention of obstetric complications, and includes little information regarding future diabetes risk [38]. After childbirth, the participants were transferred from tertiary hospitals to primary care facilities, but the staff in the latter did not know whether the women had previously experienced GDM, and therefore the continuity of maternal care, especially with regard to postpartum glucose management, was interrupted [38]. In China, the situation is similar. Healthcare professionals at maternity hospitals routinely help women who experience GDM during pregnancy and the early postpartum period to understand the severity of diabetes and the risk of developing diabetes in the future, and provide personalized health education to motivate them to make necessary lifestyle changes. In addition, maternity and primary hospitals can make use of information systems to affect a seamless handover. Specifically, they can make use of media including the telephone, email, WeChat, and other digital applications to continue the management of GDM [19]. This has been shown to be accepted by women in previous studies [19, 39], and the women in the present study expressed similar opinions.

Of particular note, in China, the traditional month-long perinatal confinement period is considered to be a barrier to women’s engagement with a healthy lifestyle. Women are usually required to undergo confinement after giving birth and there are certain contraindications with respect to diet and activities during puerperium [40]. For example, women in some areas are prohibited from eating vegetables, fruit, and cold food; are required to consume substantial amounts of chicken soup and brown sugar water; and are not permitted to go out during the confinement period. Furthermore, some of these conventions, such as drinking a lot of soup and not performing vigorous physical activity, can even last until weaning. During this period, the women are cared for in general by their mothers or mothers-in-law, who may follow traditional customs. Furthermore, the birth of a baby engenders changes in the family structure and the social roles of its members. Mothers generally have to take the bulk of the responsibility for raising their babies, which may put them under substantial physical and psychological stress and reduce their concern for their own health. Many women say that postpartum fatigue and a lack of time, owing to the necessity to feed and care for the newborn, are the major barriers to maintaining a healthy lifestyle [19, 20, 38]. The necessity to rest or sleep is considered to be more important than exercise when women have some free time, and in their new role, they often prioritize the comfort and needs of their children over their personal health [38, 41]. Moreover, when their maternity leave finishes, work and childcare occupy most of their time and energy, greatly reducing the amount of time they have available for regular exercise [19].

As reported in a systematic review, social support can improve an individual’s ability to change and maintain new lifestyle habits [42]. Similarly, the availability of adequate support has been identified as a significant element in the successful modification of the health-related behaviors of such women [18, 38]. The foremost emotional motivating factor for these women is from their families, whose encouragement provides them with a sense of love and belonging. In particular, as we have shown in the present study, spouses with a high level of health awareness often accompany women and encourage them to do physical exercise, which is regarded as a strong incentive to achieve a healthy lifestyle [38]. In addition, peers can build a mutual aid system by sharing experiences and motivating one another [43], which empowers patients with diabetes to improve their self-efficacy [44]. In the present study, some women suggested the setting up of a WeChat group to share knowledge and skills regarding diabetes, based on their own personal experiences, a finding that was also made in another qualitative study [43]. This provides a reference for us to formulate more targeted and effective strategies to assist this high-risk population.

In the present study, most of the participants had a poor understanding of health information and limited knowledge regarding the association between GDM and long-term disease risk, and these were the primary obstacles to compliance with healthy lifestyle behaviors in women after giving birth, consistent with the findings of other studies [18, 38]. Because the abnormalities in glucose metabolism disappear soon after delivery, some of the women believed that their blood glucose status had returned to normal, and therefore they resumed their pre-pregnancy lifestyle [45, 46]. This was particularly the case in the women who did not administer insulin or maintain a strict diet and exercise regimen to control their blood glucose concentrations during pregnancy. In contrast, the women who understood the risk of developing T2DM or other metabolic diseases because of online education, information provided by healthcare providers, or the experience of family members were more likely to return to the hospital for glucose screening and to change their unhealthy lifestyles [35]. In addition, knowledge of the importance of maintaining a healthy lifestyle is related to the health status of women themselves.

Women’s perception of the ease or difficulty of consuming a balanced diet, undertaking regular activity, and participating in health screening also makes a difference. We found that the poor taste of coarse grains was one of the reasons why mothers were unwilling to continue eating this food after giving birth. Especially for women whose dietary preferences conflict with the recommendations, the maintenance of a healthy diet is not easy [47, 48]. Therefore, we suggest that healthcare providers should encourage women to consult a dietitian, to develop a diverse range of recipes, taking into account their food preferences. In addition, the outcomes of the present study show that women can increase their motivation by preparing a “self-reward checklist” regarding a healthy lifestyle for the long term. This technique helps reinforce particular behaviors, and has been successfully applied to weight loss, the management of diabetes, the cessation of smoking, and broader changes in health behaviors [49].

In addition to personal and interpersonal factors, the external environment affects lifestyle. The present study was conducted during the COVID-19 pandemic, and therefore the participants were locked down at home and it was difficult for them to obtain a diverse range of foods. At the same time, the closure of gyms and sports venues restricted the activity of pregnant women and caused many of them to put on weight. A rapid review revealed that there were significant decreases in the availability and utilization of postnatal care services during and after the COVID-19 lockdown [50], owing to the postponement of non-urgent treatments and changes in the care delivered [51]. In addition to environmental changes owing to unexpected events, current societal practices are generally not attuned to people with diabetes, and specifically with respect to the provision of healthy diets. In China, very few restaurants and canteens provide special meals for people with diabetes. Instead, fast food and foods high in fat and sugar are more accessible, meaning that women face great challenges in maintaining a healthy diet. Therefore, public awareness should be raised to facilitate the provision of a more convenient and optimized eating environment for this group.

### Strengths and limitations of the present study

To our knowledge, this is the first study to explore the perceptions and attitudes, and the influences on these, regarding health-related behaviors in Chinese women with a history of GDM during their second pregnancy. We have obtained an abundance of data regarding their health-related behaviors during the inter-pregnancy period, which includes both the postpartum and preconception periods. Furthermore, the interviewees in the study had experienced the lockdown of the city owing to COVID-19, which was a unique experience for the women with a history of GDM. In addition, the combination of the inductive and deductive approaches used for the data analysis facilitated the making of synergistic findings regarding the influences on and determinants of women’s behaviors. The present study also had several limitations. First, because all the participants were recruited from one center in Shanghai, the findings may be context-specific and not readily generalizable. Second, only the views and attitudes of patients regarding their health-related behaviors were captured. Future studies should explore the views of healthcare professionals and policymakers regarding the support of women with a history of GDM, and especially regarding those who want to become pregnant again, in the Chinese context.

## Conclusions

**Fig. 2:**
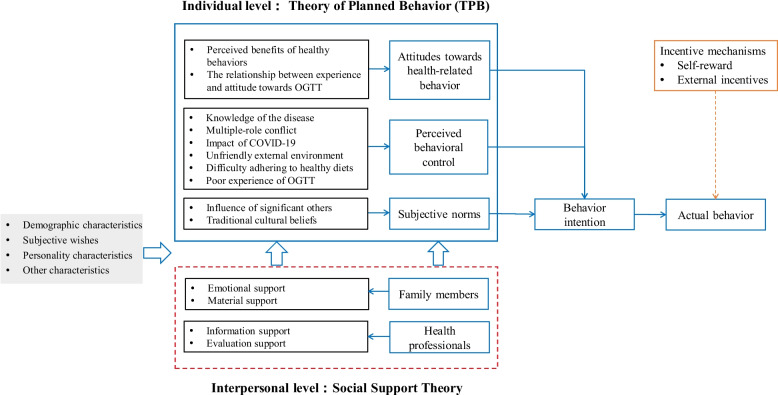
Intervention hypothesis, based on the results identified during the study. The red dotted line indicates the intervention hypothesis that is derived from the study data and the social support theory

In the present qualitative study, we have shown that health-related behaviors are affected by multiple factors in women with a history of GDM. As shown in Fig. 2, healthcare professionals should provide enough information regarding the disease and help women, as well as their families, to understand the associated future risks, to facilitate the development of a positive attitude toward healthy behaviors. In addition, the power of family and other social support networks to improve women’s subjective norms, as well as their intentions to actively adopt a healthy lifestyle, should be taken into account.

## Data Availability

The data that support the findings of this study can be obtained by contacting the corresponding author Na Wang, upon reasonable request.

## Abbreviations

GDM: Gestational diabetes mellitus
T2DM: Type 2 diabetes mellitus
OGTT: Oral glucose tolerance testing
TPB: Theory of planned behavior
